# Morphologic alterations of the fear circuitry: the role of sex hormones and oral contraceptives

**DOI:** 10.3389/fendo.2023.1228504

**Published:** 2023-11-07

**Authors:** Alexandra Brouillard, Lisa-Marie Davignon, Anne-Marie Turcotte, Marie-France Marin

**Affiliations:** ^1^ Research Center of the Institut Universitaire en Santé Mentale de Montréal, Montreal, QC, Canada; ^2^ Department of Psychology, University of Quebec in Montreal, Montreal, QC, Canada; ^3^ Department of Medicine, University of Montreal, Montreal, QC, Canada

**Keywords:** sex hormones, oral contraceptives, structural MRI, fear circuitry, gray matter volume, cortical thickness

## Abstract

**Background:**

Endogenous sex hormones and oral contraceptives (OCs) have been shown to influence key regions implicated in fear processing. While OC use has been found to impact brain morphology, methodological challenges remain to be addressed, such as avoiding selection bias between OC users and non-users, as well as examining potential lasting effects of OC intake.

**Objective:**

We investigated the current and lasting effects of OC use, as well as the interplay between the current hormonal milieu and history of hormonal contraception use on structural correlates of the fear circuitry. We also examined the role of endogenous and exogenous sex hormones within this network.

**Methods:**

We recruited healthy adults aged 23-35 who identified as women currently using (*n* = 62) or having used (*n* = 37) solely combined OCs, women who never used any hormonal contraceptives (*n* = 40), or men (*n* = 41). Salivary endogenous sex hormones and current users’ salivary ethinyl estradiol (EE) were assessed using liquid chromatography – tandem mass spectrometry. Using structural magnetic resonance imaging, we extracted surface-based gray matter volumes (GMVs) and cortical thickness (CT) for regions of interest of the fear circuitry. Exploratory whole-brain analyses were conducted with surface-based and voxel-based morphometry methods.

**Results:**

Compared to men, all three groups of women exhibited a larger GMV of the dorsal anterior cingulate cortex, while only current users showed a thinner ventromedial prefrontal cortex. Irrespective of the menstrual cycle phase, never users exhibited a thicker right anterior insular cortex than past users. While associations with endogenous sex hormones remain unclear, we showed that EE dosage in current users had a greater influence on brain anatomy compared to salivary EE levels and progestin androgenicity, with lower doses being associated with smaller cortical GMVs.

**Discussion:**

Our results highlight a sex difference for the dorsal anterior cingulate cortex GMV (a fear-promoting region), as well as a reduced CT of the ventromedial prefrontal cortex (a fear-inhibiting region) specific to current OC use. Precisely, this finding was driven by lower EE doses. These findings may represent structural vulnerabilities to anxiety and stress-related disorders. We showed little evidence of durable anatomical effects, suggesting that OC intake can (reversibly) affect fear-related brain morphology.

## 1 Introduction

Fear is a universal and adaptative emotion (1) but excessive fear reactions can be deleterious to the psychological and social functioning of individuals (2, 3). Dysfunctions resulting from poor fear regulation characterize anxiety disorders and post-traumatic stress disorder (PTSD) (4–6), where behavioral clinical treatments rely on the concept of fear extinction (i.e., safety learning, 4, 5, 7–11). As deficits in fear extinction have been identified in these psychopathologies, impaired extinction may promote the maintenance of anxiety symptoms (12–15).

Anxiety and stress-related disorders are more common in women (3, 16) but our understanding of this elevated vulnerability is limited. This knowledge gap is in part be due to the exclusion of females and women in animal and human science. Indeed, anxiety research has been oriented towards males and men (the so-called ‘male bias’ phenomenon). As of 2012, less than 2% of fear-related publications were conducted on female brains (6). This bias remains apparent in recent research where 65% of 2021 preclinical anxiety models were studied in males only (17). From 2009 to 2019, neuroscience and psychiatry publications showed a considerable increase in the inclusion of both sexes, although the proportion of male-only papers did not decrease. In fact, male-only manuscripts are 9 times more common than female-only papers in neuroscience and psychiatry journals (18). Female underrepresentation is mainly driven by the assumption that sex hormone fluctuations would lead to increased variability in results, albeit this argument has not been empirically supported (17, 19, 20).

### 1.1 Modulation of the fear circuitry by sex hormones

Scientific attention allocated to topics concerning women enabled researchers to connect endocrine activity to anxiety. Indeed, paradigms using naturally-fluctuating hormones (e.g., estradiol [E2] and progesterone [P]) or synthetic hormones (e.g., oral contraceptives [OCs]) have revealed that sex hormones modulate anxiety behaviors (for reviews see 16, 21, 22). Endogenous sex hormones are the end result of the endocrine cascade of the hypothalamic-pituitary-gonadal (HPG) axis and can fluctuate according to the menstrual cycle. That said, used by more than 150 million women worldwide (23), OCs act as a powerful disruptor of the menstrual cycle. Combined OCs (COCs) are the most common type and are made up of synthetic estrogen (e.g., ethinyl estradiol [EE]) with a progestin (e.g., levonogestrel). These exogenous hormones bind to estrogen and P receptors in brain regions involved in the HPG axis, thereby suppressing sex hormone secretion via negative feedback (24–26). Additionally, EE induces hepatic synthesis of the sex hormone binding globulin (SHBG), reducing not only free (unbound) E2 and P levels but also free testosterone (T) (27).

Notably, lowered endogenous E2 levels (either naturally occurring during the early follicular phase of the menstrual cycle or induced by OC use) is a key contributor to fear maintenance as evidenced using fear conditioning and extinction protocols (28–30). At the neural level, sex hormones are known to modulate the brain network implicated in fear processes (28, 31–34). Major brain regions of the fear circuitry include the amygdala, hippocampus, hypothalamus, anterior insular cortex (AIC), dorsal anterior cingulate cortex (dACC), rostral anterior cingulate cortex (rACC), and ventromedial prefrontal cortex (vmPFC) (32, 35–40). Particularly, the amygdala, hippocampus, and vmPFC express high levels of sex hormone receptors and as such, provide insight into how sex hormones exert their influence on fear (6, 28, 41, 42).

Fluctuations in sex hormones have been shown to influence brain activity of the fear circuitry (for reviews see 28, 31, 32). Human neuroimaging studies have associated high E2 with increased activations within this network (33, 43, 44), though fewer studies have investigated the role of sex hormones on its anatomy. The most consistent observation of the latter is the trophic anatomical effects of E2 in the hippocampus (45–48). Although less consistently reported, morphologic changes of fear-related regions have been reported throughout the menstrual cycle (e.g., changes in the dACC (49), AIC (50), and amygdala (51)). OC use has also been associated with a reduction of the hippocampus (52–54), amygdala (55), hypothalamus (56), and prefrontal cortical thickness (57, 58). Additionally, ‘acute’ sex hormone concentrations have been shown to correlate with brain structures (59). For instance, fear-related brain regions have been associated with salivary or serum E2 and T in various hormonal profiles (i.e., OC users, naturally cycling [NC] women, men) (49, 53, 57). Given the scarce literature on the topic and lack of replicability, these associations call for further empirical support. Nevertheless, these findings collectively support the involvement of endogenous and exogenous sex hormones on key regions implicated in fear processing and regulation, as well as their ability to engender significant structural alterations within this network.

### 1.2 Challenges in studying oral contraceptives

The impact of OCs on brain morphology has mainly been studied by comparing current users to NC women (according to specific phases of the menstrual cycle or not) and men (for reviews see 59–61). One of the most important methodological limitations in this type of design is the sample selection bias. Indeed, a ‘survivor effect’ may linger in samples of current OC users, knowing that women for whom the pill does not cause adverse effects are more likely to continue using it (62). Discontinuation of OCs has also been associated with psychiatric symptoms or disorders (63, 64), neuroticism (65), and mood-related side effects (66). Thereby, NC women that are previous OC users could constitute their own group that is distinct from never and current users. However, a recent investigation reported negligible differences between current, past, and never users in sociodemographic variables and personality in a matched Eastern European sample (67). Nonetheless, given that the study of OCs is normally done using cross-sectional designs between users and non-users, careful attention must be paid to the choice of comparison groups.

The vast majority of research on OCs has focused on acute (i.e., ‘here and now’, activational) effects underlying its use. Yet, hormonal events have been shown to exert long-lasting neural changes, as reported in the context of puberty (68, 69), pregnancy (70–72), and menopausal hormonal therapy (73, 74). Regarding OC use, a paucity of studies distinguishing current, past, and never users suggests that OC intake could lead to long-term effects on endogenous sex hormone concentrations (75), brain structures (76, 77), cognition (78, 79), and anxiety symptoms (as experienced during the COVID-19 pandemic, 80). It is still unclear whether such lasting effects exist and if so, by which mechanisms OCs exert their influence after cessation. Still, it appears obvious that the simple comparison of OC users and NC women would generate its share of heterogeneity, as grouping NC women would most likely comprise past and never users.

Generally, studies have homogenized their subset of OC users by selecting COC users only (i.e., excluding progestin-only OC users). If there has been a keen interest over time to refine methodologies to the specific use of COCs, very few studies have considered women’s lifetime history of hormonal contraceptives. Yet, as previously stated, an increasing body of research points to differential effects between current users, past users, and never users (77, 78, 80–82). Knowing that women commonly try several types of hormonal contraceptives in their lifetime, this factor may introduce unwanted noise. Therefore, when examining current and past COC users, rigorous methodological standards can be achieved by considering the history of hormonal contraceptive use. To our knowledge, only two studies have considered contraception history and restricted their sample to women who had only used OCs or COCs throughout their lifetime (76, 83).

Relatedly, COCs is a broad category composed of more than 30 formulations with varying doses and/or synthetic compound compositions (24, 84). Doses of EE range from 10 to 50μg, although COCs containing 35μg of EE or less are the most commonly used. While EE is typically the estrogenic constituent in COCs, progestins (and their dosage) are heterogenous and differ according to their chemical structure and pharmacodynamics (85). Androgenic activity is commonly used to classify progestins and as such, can be classified as high (e.g., levonogestrel), low (e.g., norgestimate, desogestrel), or anti-androgenic (e.g., drospirenone, cyproterone acetate) progestins (24, 86). Of note, androgenicity effects have been examined on cognitive functions (87–91) and neural correlates (79, 82, 83, 92). Anatomically, a larger volume of the bilateral fusiform face area was found in anti-androgenic users compared to androgenic users, while EE dose did not correlate with this region (82). Furthermore, the effects of exogenous hormone concentrations on brain structures have not been reported to date. Synthetic compounds (e.g., EE) are not captured by traditional immunoassays of circulating E2 in serum or saliva, meaning that the current literature has only partly depicted the endocrine activity of OC users (84).

Finally, many metrics to quantify brain morphology have emerged over the years. While this represents a fundamental strength for the field of neuroscience, it has also inevitably led to heterogeneous findings. Structural imaging studies have used different measures to assess brain tissues such as gray or white matter volume, cortical thickness (CT), cortical surface area, and folding/gyrification (61, 93, 94). Voxel-based morphometry (VBM) has traditionally been the most popular method to quantify gray matter volume (GMV), though it can also be measured using surfaced-based morphometry (SBM; as the product of CT and surface area for cortical GMV). However, it is well known that changes in GMV are unspecific and can result in differing interpretations (e.g., changes in CT, folding, or surface area of the cortex) (61, 95). Interestingly, GMV is the most frequent metric used to quantify OC changes in the brain (61) and only one group has investigated the impact of OCs on other gray matter (GM) phenotypes, namely CT (57, 58).

Despite the growing interest in studying women’s health, much remains to be discovered about the impact of sex hormones and OCs on the brain, cognition, behavior, and mental health. In psychoneuroendocrinology, there is a lack of consensus with regard to the effects of sex hormones and OCs, mostly due to the relatively small number of studies and heterogeneous methodologies used (96–102). This study aimed to investigate the current and potentially lasting effects of COC use, as well as the role of endogenous and exogenous sex hormones on structural correlates of the fear circuitry. We recruited women currently using or having used solely COCs in their lifetime, women who never used any hormonal contraceptives, and men. Using structural magnetic resonance imaging (MRI), we extracted GMV and CT of key regions of the fear circuitry. Comparing current users, past users, never users, and men allowed us to examine whether COC use was associated with acute (i.e., current users vs. never users = past users) or long-term (i.e., current users = past users vs. never users) morphologic alterations, as well as to detect sex differences. Additionally, we conducted analyses to explore the associations between endogenous sex hormones and brain morphology in past users, never users, and men, as well as with EE (salivary levels and prescribed doses) and androgenicity in current COC users.

## 2 Materials and methods

### 2.1 Participants and procedure

This study stems from a larger research project investigating the neural, cognitive, and endocrine correlates of COC use, which involved three laboratory sessions. Participants were all healthy adults aged between 23 and 35 years old. This minimal age criterion was set to limit brain variability due to late-adolescence maturation (103, 104) without compromising recruitment feasibility (i.e., undergraduates were still allowed to apply). According to their hormonal profile and COC history, participants were divided into groups of men, never users, past users, and current users. Eligibility was confirmed after a thorough phone screening, ensuring that participants from all groups met the following inclusion/exclusion criteria: French speakers, no past or current medical/psychological diagnosis, no current or past pregnancy, no usage of medication affecting the endocrine system (other than COCs), and no regular usage of drugs and alcohol. Participants also completed an MRI screening questionnaire for safety purposes (e.g., no metallic implant, claustrophobia). For COC criteria, past COC users were required to have used their COC and have stopped it for over a year (*M_duration_* = 6.74 years ± 3.21; *M_cessation_* = 3.37 years ± 2.15). Current COC users had to be using it for at least 3 months (*M* = 8.97 years ± 3.83). Regarding contraception history, past and current users must not have used other types of hormonal contraceptives than COCs in their lifetime (i.e., progestin-only pill, intra-uterine device, patch, vaginal ring, injection, or implant). Never users must not have used any hormonal contraceptives in their lifetime. As for NC women, never users and past users were expected to have a regular menstrual cycle (*M* = 29.26 days ± 3.01; normal cycle length of 21-35 days, 105). As these women monitored their menstrual cycles, laboratory sessions were scheduled during either the early follicular phase (day 1-5 of the menstrual cycle, where day 1 corresponds to the onset of menses) or the pre-ovulatory phase (1-4 days before ovulation, where ovulation day was calculated as average cycle length minus 14). One never user had a cycle length longer than 35 days and two women (one past user, one never user) were not able to quantify their cycle length, they were therefore scheduled during the early follicular phase. Given that E2 has been found to have a marked influence on the fear circuitry (see section 1.1.), these two cycle phases were selected due to their distinct E2 concentrations. This methodological detail allowed us to investigate the role of cycle phases in NC women, as well as to control for hormone levels within this group of women (i.e., equivalent distributions of cycle phases between both groups).

Data for the present manuscript were collected during the second session of the larger study. Before entering the MRI scanner, saliva samples were collected for later quantification of sex hormone levels. In the MRI scanner, participants first underwent a structural sequence before going through a task for functional sequences. All sessions were scheduled between 8:15AM and 7PM. This study was reviewed and approved by the research ethics board of the *Centre intégré universitaire de santé et de services sociaux de l’Est-de-l’Île-de-Montréal*. Individuals provided written consent before participating in this study, had the right to terminate the experiment at any time, and were offered monetary compensation for their time. A total of 181 participants completed the procedure for the present study.

### 2.2 Questionnaires

The following questionnaires were completed by participants to evaluate potential confounds between our groups and on structural outcomes. Importantly, these constructs have all been associated with COC use and/or brain morphology (65, 106–111). Participants completed the 1) Beck Depression Inventory-II (BDI), a 21-item questionnaire assessing physiological and psychological symptoms related to depression (range 0-63, 112, 113), 2) Brief Trauma Questionnaire (BTQ), a 10-item questionnaire derived from the Brief Trauma Interview (114) that evaluates previous exposure to stressful/traumatic events (115), 3) Neuroticism scale from the NEO-Five Factor Inventory-3, a 12-item scale measuring the tendency towards negative affect and emotional instability (range 12-60, 116), 4) Santa Clara Strength of Religions Faith Questionnaire (SCSRFQ), 10 questions regarding religiosity (range 10-40, 117), and 5) trait form of the State and Trait Anxiety Questionnaire (STAI-T), which measures anxiety as a personality trait through 20 items (range 20-80, 118, 119). All questionnaires (except the BDI) were answered online on Qualtrics XM between the first and second sessions of the larger study. The BDI was completed at the end of the third session and participants scoring ≥ 14 (i.e., mild depression symptomatology) were offered psychological resources.

### 2.3 Sex hormone assessment

Salivary sex hormone concentrations were collected using the passive drool method. A 2mL sample was provided in a salivette by all participants and stored temporarily in a -20°C freezer at the MRI facility. Within a period of 2 months, samples were transferred to a -80°C freezer at our research center for long-term conservation. Upon the time of analysis, samples were shipped with dry ice to ZRT Laboratory (Beaverton, Oregon, USA), a lab specialized in salivary assays of sex hormones using liquid chromatography – tandem mass spectrometry (LC-MS/MS). Assays were run with an AB Sciex Triple Quad 5500 system. Saliva was mixed with internal standards, then extraction of steroids was performed by Cl 8 column chromatography. Steroids were eluted from the solid phase extraction and dried under nitrogen. Derivatization was carried out on dried samples (120), after which they were diluted and injected for LC-MS/MS analysis with analytical separation performed on an Agilent Poroshell 120 EC-C8 column and ionization by atmospheric pression chemical ionization (121). The lower limit of quantification (LLOQ) was 0.30 pg/mL for E2, 5 pg/mL for P, 3 pg/mL for T, and 0.40 pg/mL for EE. Given that we had data on which participants were expected to truly have circulating EE levels (i.e., COC users in the active phase of their regimen), we used the lower limit of detection (LOD) of 0.11 pg/mL for EE values below the LLOQ.

### 2.4 MRI data acquisition

Structural images were acquired on a Siemens Magnetom Prisma 3T scan using a 64-channel coil. A T1-weighted Multi-Echo Magnetization-Prepared Rapid Gradient-Echo (MEMPRAGE) sequence was used to collect 176 high-resolution images (TR = 2530ms, TE = 1.62ms, FOV = 176mm, flip angle = 7°, voxel size = 1mm^3^). Visual inspection for motion artifacts was performed on raw data for each participant based on a 4-point scale protocol (122; https://github.com/CoBrALab/documentation/wiki/Motion-Quality-Control-(QC)-Manual). This procedure resulted in the exclusion of one participant (past COC user).

### 2.5 Surface-based morphometry

Raw anatomical images were processed using FreeSurfer (http://surfer.nmr.mgh.harvard.edu). The recon-all pipeline of Freesurfer 6.0 was performed on CBRAIN, an online open-source software (123) and allowed for the automatic generation of a 3D cortical surface model. Segmentation of white matter (WM) and GM (pial) surfaces was visually inspected for every participant. No major flaws were detected by visual inspection (e.g., WM/pial surface segmentation into the skull or ventricles).

We extracted both GMV and CT phenotypes for regions of interest (ROI)-based analyses. The Destrieux atlas (124, 125) was used for the parcellation of cortical ROIs. To select precise parcels of the fear circuitry, we inspected the brain map for the term ‘fear’ on Neurosynth (neurosynth.org), an online platform for the automated synthesis of functional MRI data. Using peak coordinates, we identified which Destrieux’s labels overlapped with fear-related clusters. The dACC was best delimited by the anterior part of the middle cingulate gyrus and sulcus (aMCC, label#7), which was further validated by Vogt et al. (126) as being involved in fear expression. We defined the rACC as the anterior part of the cingulate gyrus and sulcus (ACC, label #6). For the AIC, we combined the short insular gyrus (label #18) and anterior segment of the circular sulcus of the insula (label #47). Finally, the vmPFC was defined as the sum of the straight gyrus (gyrus rectus, label #31) and suborbital sulcus (label #70). At the subcortical level, GMV of the amygdala and hippocampus were obtained from FreeSurfer’s segmentation. The hypothalamus was segmented using the automated tool of Billot et al. (127). GMV of the whole structure was used. All ROIs were extracted by hemisphere. Exploratory vertex-by-vertex whole-brain analyses (WBA) were conducted for cortical GMV and CT using a smoothing full-width half maximum (FWHM) kernel of 10mm and 20mm, respectively.

### 2.6 Voxel-based morphometry

Additionally, we performed VBM as a secondary analytic method. Data were analyzed using Matlab (R2018b, Mathworks Inc.) and SPM12 (http://www.fil.ion.ucl.ac.uk/spm). Images were reoriented along the anterior-posterior commissure line and the anterior commissure was set as the coordinate origin. Images were segmented into GM, WM, and cerebrospinal fluid (Ashburner and Friston, 2005). The Diffeomorphic Anatomical Registration Through Lie Algebra (DARTEL) algorithm was used for estimating deformations and increasing the accuracy of between-subjects alignment (Ashburner, 2007). Images were modulated and spatially normalized to the Montreal Neurological Institute (MNI) space. Normalized GM images were smoothed with a FWHM kernel of 8 mm. GM voxels with a value < 0.2 were excluded from statistical analyses. Global normalization was set at proportional scaling to account for TIV.

### 2.7 Analytic approach

In the present study, we examined two phenotypes of the GM, namely volume and thickness. As volume is an unspecific feature of the GM (95), we also measured CT to refine the interpretation of the results. Further, we used two volumetric methods to generate surface-based and voxel-based GMV. Given the well-documented difficulty of low reproducibility in MRI studies (128–130), this motivated our team to conduct analyses not only in FreeSurfer but in SPM using VBM to evaluate the robustness of analytical variability of GMV results. As such, between-software convergence would strengthen confidence in the obtained results (130). Moreover, given that FreeSurfer’s volumetric WBA is restricted to the cortex, the use of SPM’s VBM allowed us to examine cortical, subcortical, and cerebellar voxels.

Regarding endocrine quantification, the use of LC-MS/MS resulted in a considerable rate of levels falling under the LLOQ. Left-censored undetected values were high for E2 (< 0.3 pg/mL: 110/180, 61.1%), P (< 5 pg/mL: 125/180, 69.4%), and moderate for T (< 3 pg/mL: 51/180, 28.3%). This small proportion of detected values was also reported with LC-MS/MS for salivary E2 (131). Nonetheless, undetected values followed a coherent pattern with nearly all of them being distributed in expected low hormonal profiles. Frequencies of undetected E2 were distributed as follows: 57 COC users, 27 NC women in the early follicular phase, and 23 men (107/110, 97.27%). Given that the luteal phase of the menstrual cycle was not targeted in NC women, all our participants were recruited in a low state of P. This provides insight into the high undetected P rate in every group (≥ 64.5%). For T, 39 COC users, 11 early follicular NC women, and 1 pre-ovulatory NC woman accounted for the undetected values. For COC users in the active phase of their regimen (*n* = 55), EE levels were below the LOD for 8 women (14.5%). Given that our missing values were not missing at random (NMAR) and as recommended by Herbers et al. (132), we addressed this issue with multiple imputation from a fitted lognormal distribution using their same procedure implemented in R with the fitdistrplus and EnvStats packages (133, 134; the code used by Hebers et al. is accessible at https://osf.io/spgtv/). This imputation method was found to be valid, despite the high proportion of missingness in left-censored datasets (135). Using the SPSS toolbox, we generated 10 datasets with simulated values between 0.01 pg/mL and the LLOQ for each endogenous hormone (e.g., 0.01-0.29 pg/mL for E2). The LOD was used as the upper bound for EE. For statistics, we reported the mean value of each metric (e.g., *F_M_* = average of the 10 *F*s). Due to highly unbalanced distributions and to be coherent with the imputation procedure performed, we then log-transformed endocrine variables. Finally, outliers were examined. Z-scores were derived for men, early follicular NC women, pre-ovulatory NC women, and COC users on log-transformed variables. Using a criterion of ≥ ± 3.29 (136, 137), we identified two outliers for E2 (COC users), one outlier for P (COC user), two outliers for T (pre-ovulatory NC woman and man), and one outlier for EE. All outliers were winsorized with respect to groups, using the next highest value of each group (138) on the imputed log-transformed variables. Additionally, we removed endocrine values for one participant (man) due to an invalid assessment (technical issue/low internal standard). For descriptive purposes only, we winsorized imputed raw (untransformed) values and reported endocrine concentrations in pg/mL.

All ROI analyses were performed using SPSS 27 (IBM). We conducted general linear models (GLMs) for each ROI with Group (men, never users, past users, current users) as the between-subjects factor and Hemisphere (left, right) as the within-subjects factor. We controlled for age for both GMV and CT analyses, as well as for TIV in GMV analyses (for a justification, see 139, 140). Given our between-group design, we examined further potential confounders thereafter. Covariates were selected from analyses performed on various sociodemographic and psychological outcomes with a threshold set at *p* <.10. First, study groups were compared using ANOVAs and chi-square tests for continuous and categorical variables, respectively. Second, correlations were performed between potential confounders and TIV/global CT. Using the ‘disjunctive cause criterion’ approach for covariate selection (141), we selected variables that were either associated with the study groups, brain morphology (TIV/global CT), or both. Thus, we decided to initially run the minimally adjusted models (e.g., age and TIV for GMV) and re-run the fully adjusted models for additional confounders (e.g., ethnicity, relationship status, religiosity). To investigate the interplay between current dynamics of the menstrual cycle and history of COC use, we focused on both groups of NC women (namely, never users and past users, as well as the cycle phase they were in during data collection). Accordingly, we performed Group (past users, never users) x Cycle phase (early follicular, pre-ovulatory) GLMs. Similar to the previous model, we implemented the same covariate selection plan.

To investigate the impact of endogenous sex hormones, we re-ran previous Group x Hemisphere GLMs for each ROI with added hormonal concentrations. Given that these specific analyses relied on the continuous nature of hormonal data, we excluded variables showing a proportion of imputation higher than 60%. This procedure led to the exclusion of COC users in this set of analyses and P levels for the three remaining groups (i.e., men, never users, past users). Conceptually, this decision is supported by the fact that COC users should be in an endogenous ‘shut-down’ state and that P variability was expected to be minimal across participants (due to the nature of our study design). As such, models were built to explore the effects of Group (3) x Hemisphere (2) x E2 and Group (3) x Hemisphere (2) x T, all while controlling for age (and TIV for GMV). In a second step, significant results were re-examined by running a sensitivity analysis with subcategorized hormonal data (e.g., ‘low levels’ = undetected imputed values, ‘medium levels’ = detected but low values, ‘high’ = detected and higher values). This was performed to assess the robustness of the results obtained with multiple imputations. No WBA was performed for this analysis subset given that multiple imputation could not be implemented in FreeSurfer and SPM.

In COC users, we explored the role of exogenous hormones as assessed by salivary EE levels or EE prescribed dose (contained in each pill, in μg) and androgenicity. As EE levels were accounted for, EE models were restricted to women tested in the active phase of their COC (*n* = 55). Among them, 48 used monophasic regimens, while seven used triphasic regimens with a constant dose of EE across all active pills. Given the few and unevenly distributed data points for EE dose (as reported in **Table 1**), we grouped users into low (10-25μg) or high (30-35μg) dosages. In our sample, no COC users used 50μg of EE. For each ROI, EE GLMs were tested with Hemisphere (left, right) x EE dose (low, high) and Hemisphere x EE levels as variables of interest, where age and TIV (for GMV only) were used as covariates. Of note, we did not investigate the impact of lifetime EE dose in current users or previous EE dose within past users given that these calculations led to insufficient sample sizes (i.e., 33/62 current users and 14/37 past users having used the same dosage over lifetime use). As for progestin androgenicity, we classified users into high (*n* = 27), low (*n* = 18), and anti-androgenic (*n* = 17) COCs categories (24) (see **Table 1**). The influence of androgenicity was explored with Hemisphere (right, left) x Androgenicity (anti, low, high) GLMs, while controlling for age and TIV (for GMV only). Considering the small sample size, we first ran separate models for EE and progestin androgenicity. However, EE dosage and progestin androgenicity can vary within formulations and potentially interact with each other. Indeed, the chi-square test showed that a higher proportion of low EE doses were classified as high androgenic COCs, while high EE doses were more likely to be classified as low androgenic COCs (*p* = .004). Therefore, in a second step, we adjusted for one or the other in their respective set of analyses. Although statistically underpowered, this allowed us to verify the stability of our results when covarying out an important confounder.

**Table 1:** Sample characteristics for current users, past users, never users, and men.

Variable	Current users (*n* = 62)	Past users (*n* = 37)	Never users (*n* = 40)	Men (*n* = 41)	*P*-value
Potential confounders					
TIV (L)	1.51 (0.12)	1.50 (0.09)	1.48 (0.11)	1.68 (0.12)	**<.001**
Global CT (mm)	2.52 (0.07)	2.52 (0.06)	2.52 (0.07)	2.54 (0.07)	.272
Age	26.31 (2.88)	27.57 (3.30)	26.13 (3.16)	26.27 (3.59)	.169
Ethnicity					
*Caucasian*	46 (74.2%)	29 (78.4%)	21 (52.5%)	27 (65.9%)	**.059**
*Other*	16 (25.8%)	8 (21.6%)	19 (47.5%)	14 (34.1%)	
Mother tongue					
*French*	47 (75.8%)	33 (89.2%)	27 (67.5%)	29 (72.5%)	.101
*Other*	15 (24.2%)	4 (10.8%)	13 (32.5%)	11 (27.5%)	
Years of education	18.05 (2.03)	17.55 (2.20)	17.08 (2.04)	17.10 (2.32)	**.072**
Body mass index	22.95 (2.90)	23.53 (3.50)	23.57 (4.61)	23.96 (3.73)	.572
Relationship status					
*In a relationship*	40 (65.6%)	16 (44.4%)	10 (26.3%)	12 (30.0%)	**<.001**
*Single*	21 (34.4%)	20 (55.6%)	28 (73.7%)	28 (70.0%)	
Practicing physical activity					
*Yes*	53 (85.5%)	26 (70.3%)	32 (80.0%)	34 (82.9%)	.306
*No*	9 (14.5%)	11 (29.7%)	8 (20.0%)	7 (17.1%)	
Religiosity (SCSRFQ)	13.92 (7.18)	14.35 (7.33)	17.38 (9.37)	16.03 (8.37)	.151
Neuroticism (NEO-FFI-3)	35.55 (8.79)	36.65 (8.52)	36.13 (8.23)	33.18 (9.34)	.307
Trait anxiety (STAI-T)	40.89 (10.39)	39.08 (8.80)	40.55 (7.25)	39.40 (10.07)	.756
Depressive symptoms (BDI)	8.15 (6.13)	7.83 (5.54)	8.95 (7.49)	7.74 (6.43)	.841
Adverse events (BTQ)	1.10 (1.05)	1.24 (1.19)	1.30 (1.49)	0.95 (1.24)	.580
Hormone levels					
Estradiol (pg/mL)	0.19 (0.11)	0.56 (0.63)	0.46 (0.44)	0.26 (0.14)	**<.001**
Progesterone (pg/mL)	5.65 (10.05)	4.60 (4.78)	4.56 (5.01)	5.26 (5.78)	.837
Testosterone (pg/mL)	2.23 (1.21)	4.13 (1.99)	4.25 (2.41)	53.58 (20.98)	**<.001**

For TIV, CT, age, years of education, body mass index, religiosity, neuroticism, trait anxiety, depressive symptoms, and adverse events, data represent group means (SD). For ethnicity, mother tongue, relationship status, and practicing physical activity, data represent group N (group %). Bold characters indicate covariates from potential confounders selected for fully adjusted models based on a threshold set at p <.10 (hormone levels were not considered as covariates). For psychological measures (religiosity, neuroticism, trait anxiety, adverse events), mother tongue, and endocrine concentrations, data for one man are missing. For the depressive symptom assessment, data are based on 61 current users, 36 past users, 38 never users, and 40 men./TIV, total intracranial volume; CT, cortical thickness; SD, standard deviation.

For ROI-based analyses, significance levels were set at *p* <.05. Trend-level results were defined as.05 ≤ *p* ≤.08. Bonferroni corrections were applied to *p*-values in *post hoc* analyses. Based on the novelty of the study, uncorrected results were presented for multiple comparisons; however, adjustment for multiple comparisons was applied for the seven GMV ROIs and four CT ROIs using the False Discovery Rate (FDR) correction (142). For exploratory volumetric and thickness WBA in FreeSurfer, the Monte Carlo Null-Z Simulation was carried out to control for multiple comparisons (10 000 iterations, cluster-forming two-tailed *p* <.05, cluster-wise *p* <.05). A Bonferroni correction was applied as we tested both hemispheres. For VBM, we used a primary uncorrected threshold of *p* <.001 with a cluster extent of *k* > 10 voxels and secondary cluster-level FDR-corrected threshold of *q* <.05. Groups were compared using a *F*-test orthogonal contrast. Effect sizes were reported as partial eta squared (*η_p_^2^*). Error bars in figures denote ± standard error of the mean.

## 3 Results

### 3.1 Preliminary analyses

**Figure 1:**
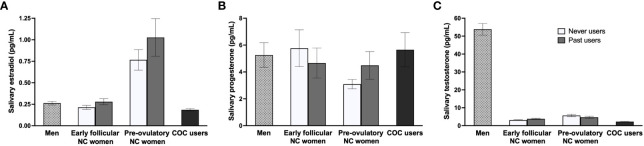
Sex hormone profiles across study groups. **(A)** As expected, pre-ovulatory naturally-cycling (NC) women had higher estradiol concentrations compared to the three other groups. Estradiol levels were similar for early follicular NC women, men, and combined oral contraceptive (COC) users. **(B)** Groups did not differ in progesterone levels. **(C)** For testosterone, men showed higher concentrations than the three groups of women, pre-ovulatory NC women exhibited greater levels than early follicular NC women, and both NC groups had more testosterone levels than COC users. No differences were found between never users and past users across the three hormones.

To account for potential diurnal variations in sex hormone levels (143–145), we examined whether the timing of study sessions, which ranged from 8:15AM to 7PM as a matter of feasibility, influenced endocrine concentrations. Our analysis revealed no significant influence of session timing (AM/PM) on hormone levels within each group (*p* ≥.130). Groups were also equally distributed for AM/PM testing (*p* = .190). To confirm hormonal status, GLMs were conducted on groups based on their current hormonal state. Levels of E2, P, and T were compared between men (*n* = 40), early follicular NC women (*n* = 45), pre-ovulatory NC women (*n* = 32), and COC users (*n* = 62). Analyses revealed a group difference for E2 [*F_M_*_(3, 175)_ = 34.80, *p_M_* <.001, *η_p_^2^_M_* = .369] and T [*F_M_*_(3, 175)_ = 213.98, *p_M_* <.001, *η_p_^2^_M_* = .784]. Games-Howell *post hoc* tests showed higher E2 concentrations in pre-ovulatory NC women compared to the three other groups (*p_M_* s <.001). Early follicular NC women and men had similar E2 levels (*p_M_* = .898), which were both comparable to COC users (*p_M_* = .267 and.117 respectively). For T, men showed higher concentrations than the three groups of women (*p_M_* s <.001). Pre-ovulatory NC women exhibited greater T levels than early follicular NC women (*p_M_* = .033) and both NC groups had more T levels than COC users (*p_M_* s ≤.006). Further, a Group (2) x Cycle phase (2) GLM was conducted between past users and never users. For the three endogenous hormones, no differences were found between the two groups nor for the interaction term (*p_M_* s ≥.185). For cycle phase, irrespective of groups, E2 and T were significant and followed the same pattern as in the first set of hormonal analyses. Untransformed hormonal means are reported in **Table 1**, while both sets of group analyses are summarized in **Figure 1**. In sum, the salivary endocrine assessment revealed expected variations in our study groups. Never and past users were equivalent in terms of their hormonal profile.

Sample characteristics are presented in **Table 1**. As expected, men had greater TIV than the three other groups of women (*p* <.001). No differences were observed for global CT. Regarding sociodemographic variables, men, never users, past users, and current COC users did not differ on age, mother tongue, body mass index, and physical activity practice (*p*s ≥.101). However, relationship status was disproportionately distributed across study groups (*p* <.001). Based on Bonferroni-adjusted pairwise Z-tests, current users were significantly more in relationships than the three other groups and the proportion of individuals in a relationship was higher in both current and past users than never users and men. Moreover, groups tended to differ on ethnicity (*p* = .059), where never users were less of Caucasian origin than expected. Although non-significant, a greater proportion of past users were of Caucasian origin (78.4%) than never users (52.5%) in our sample. Years of education were also marginally different between the groups (*p* = .072), although *post hoc*s did not yield significant group differences (*p*s ≥.156). Concerning psychological constructs, groups did not differ regarding religiosity, neuroticism, trait anxiety, depressive symptoms, and exposure to adverse events (*p*s ≥.151). For endocrine-related variables (**Table 2**), no differences were found for the age at menarche between the three groups of women (*p* = .498), nor for cycle phase between past and never users (*p* = .524), and reasons for COC use between current and past users (*p* = .329).

**Table 2:** Endocrine-related characteristics for current users, past users, and never users.

Variable	Current users (*n* = 62)	Past users (*n* = 37)	Never users (*n* = 40)	*P-*value
Age at menarche	12.61 (1.67)	12.43 (1.17)	12.86 (1.72)	.498
Cycle phase				
*Early follicular*		23 (62.2%)	22 (55.0%)	.524
*Pre-ovulatory*		14 (37.8%)	18 (45.0%)	
Ethinyl estradiol (pg/mL)	3.96 (15.72)			
Dose of ethinyl estradiol				
*10 μg* *20 μg* *25 μg* *30 μg* *35 μg*	1 (1.6%) 29 (16.1%) 4 (2.2%) 20 (11.1%) 8 (4.4%)			
Progestin androgenicity				
*High*				
*Levonogestrel*	27 (43.5%)			
*Low*				
*Desogestrel* *Norgestimate* *Norethindrone acetate*	13 (21.0%) 3 (4.84%) 2 (3.23%)			
*Anti*				
*Drospirenone* *Cyproterone acetate*	12 (19.35%) 5 (8.06%)			
Reason for COC use				
*Contraception* *Therapeutic/medical reasons* *Mixed reasons* *Other*	23 (37.1%) 14 (22.6%) 23 (37.1%) 2 (3.2%)	20 (54.1%) 6 (16.2%) 9 (24.3%) 2 (5.4%)		.329
Reason for stopping COCs				
*No need* *Physical side effects* *Psychological side effects* *Fear of complications* *Mixed reasons* *Other*		4 (10.8%) 6 (16.2%) 3 (8.1%) 8 (21.6) 9 (24.3%) 7 (18.9%)		
Reason for not using COCs				
*No need* *Fear of physical effects* *Fear of psychological effects* *Mixed reasons* *Other*			18 (45%) 4 (10.0%) 1 (2.5%) 12 (30.0%) 5 (12.5%)	

For age at menarche, data represent group means (SD). For menstrual cycle phase, reasons for COC use, reasons for stopping COC, and reasons for not using COC, data represent group N (group %). For the analyses related to doses of ethinyl estradiol, sample size corresponds to 55 current users in the active phase of their regimen (one user of 10 μg, 23 users of 20 μg, four users of 25 μg, 20 users of 30 μg, and seven users of 35 μg)./COCs, combined oral contraceptives; SD, standard deviation.

Correlations between TIV/global CT and potential confounders across the whole sample are reported in **Table 3**. According to the disjunctive cause criterion (141), fully adjusted models for GMV included age (conceptually), TIV, ethnicity, years of education, relationship status, and religiosity, whereas age, ethnicity, years of education, relationship status, and practicing physical activity were controlled for in fully adjusted CT models. When restricted to past users and never users, fully adjusted models included age and TIV (conceptually), mother tongue, body mass index, ethnicity, relationship status, religiosity, and adverse events for GMV analyses and age, mother tongue, body mass index, ethnicity, relationship status, and neuroticism for CT analyses (data not shown).

**Table 3:** Associations between total intracranial volume, global cortical thickness, and potential confounders across the whole sample.

	TIV		Global CT		*N*
	Coefficient	*P*-value	Coefficient	*P*-value	
Age	-0.087	.248	**-0.279**	**<.001**	180
Ethnicity	0.112	.135	**0.183**	**.014**	180
Mother tongue (French/other)	-0.006	.937	0.042	.574	179
Years of education	**-0.129**	**.085**	-0.1	.183	180
Body mass index	0.031	.679	-0.112	.135	180
Relationship status (Relationship/Single)	**-0.139**	**.067**	-0.058	.443	175
Practicing physical activity (Yes/No)	0.111	.139	**0.143**	**.056**	180
Religiosity (SCSRFQ)	**-0.148**	**.049**	-0.029	.698	179
Neuroticism (NEO-PPI-3)	-0.113	.132	-0.059	.433	179
Trait anxiety (STAI-T)	-0.042	.577	-0.058	.442	179
Depressive symptoms (BDI)	-0.087	.257	-0.003	.964	173
Adverse events (BTQ)	-0.085	.255	-0.002	.979	179
Age of menarche	0.115	.182	-0.03	.728	137
Cycle phase (Follicular/Pre-ovulatory)	0.07	.545	-0.056	.626	77

Bold characters indicate covariates selected for fully adjusted models based on a threshold set at p <.10./TIV, total intracranial volume; CT, cortical thickness.

### 3.2 Main analyses

#### 3.2.1 Are there acute or long-term effects of COCs on structural correlates of the fear circuitry?

**Figure 2:**
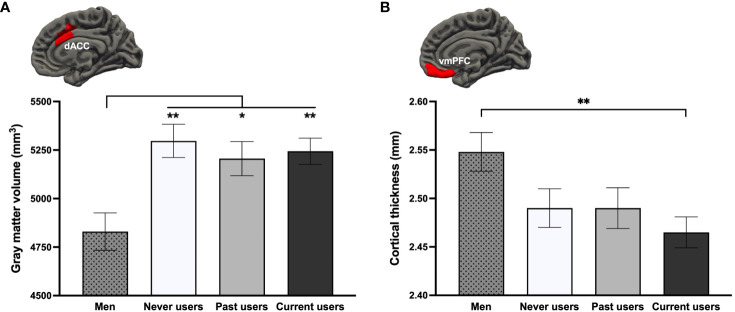
Group differences on the **(A)** bilateral dorsal anterior cingulate cortex (dACC) and **(B)** bilateral ventromedial prefrontal cortex (vmPFC) based on a region-of-interest approach. **p <.01, *p <.05.

To examine the acute or long-term effects of COC use, we performed Group (4) x Hemisphere (2) GLMs. In ROI-based GMV analyses controlling for age and TIV, a main effect of Group was observed for the dACC [*F*_(3,174)_ = 4.63, *p* = .004, *η_p_^2^* = .074, *q*FDR = .027], with all three groups of women exhibiting a larger bilateral volume than men (*p*s ≤.037; **Figure 2A**). In ROI-based CT analyses controlling for age, groups also differed regarding vmPFC thickness [*F*_(3,175)_ = 3.71, *p* = .013, *η_p_^2^* = .060, *q*FDR = .051]. Current COC users, but not past nor never users (*p*s ≥.229), had thinner bilateral vmPFCs than men (*p* = .007; **Figure 2B**). Results remained the same using fully adjusted models.

We found no significant clusters in volumetric and thickness WBA. However, SPM’s VBM revealed several clusters surviving multiple comparisons at the cluster level. Results are presented in **Table 4** and visually represented in **Supplementary Figures 1**–**3**.

**Table 4:** Voxel-based morphometry clusters emerged between the four groups using a whole-brain approach, adjusted for age and scaled for total intracranial volume.

Brain region	MNI coordinates *x y z*	*k* voxels	Peak-level *F*-value	Cluster-level *q*FDR	*Post hoc*s	Fully adjusted model cluster-level *q*FDR
Right inferior occipital gyrus/pole	48 -80 -21	3509	24.19	<0.001	M > 3 groups of women C > P_#_	<.001
Left dACC	-4 9 40	12376	16.52	<0.001	3 groups of women > M	<.001
Right postcentral gyrus	38 -21 40	2417	16.44	<0.001	3 groups of women > M	<.001
Right cerebellum	6 -75 -24	2263	13.02	<0.001	P > 3 groups	<.001
Right inferior frontal sulcus (dlPFC)	45 39 14	789	11.97	0.012	3 groups of women > M	.298
Right thalamus	2 -14 4	1167	11.24	0.002	C > M_#_	.058
Right caudal middle frontal gyrus	46 15 38	544	10.88	0.037	C = P > M	.182
Left Opercular inferior frontal gyrus	-46 14 26	1239	10.78	0.002	3 groups of women > M	.009
Left cerebellum	-42 -51 -44	1247	9.01	0.002	P > 3 groups	.010
Right superior temporal sulcus	42 -45 4	1041	8.74	0.004	N = C > M	.032
Left temporal pole/parahippocampal gyrus	-22 9 -33	593	8.73	0.033	C = P_#_ > M	.767
Right orbital gyrus	20 28 -22	577	8.53	0.033	C > N = M	.491
Left cuneus	-2 -69 10	415	8.14	0.073	C > M _(NS)_	.491

# represents post hoc comparisons that were trending towards significance./dACC, dorsal anterior cingulate cortex; dlPFC, dorsolateral prefrontal cortex; M, Men; N, Never users; P, past users; C, Current users; NS, non-significant.

#### 3.2.2 Does the current hormonal milieu interact with the history of contraception use in NC women?

**Figure 3:**
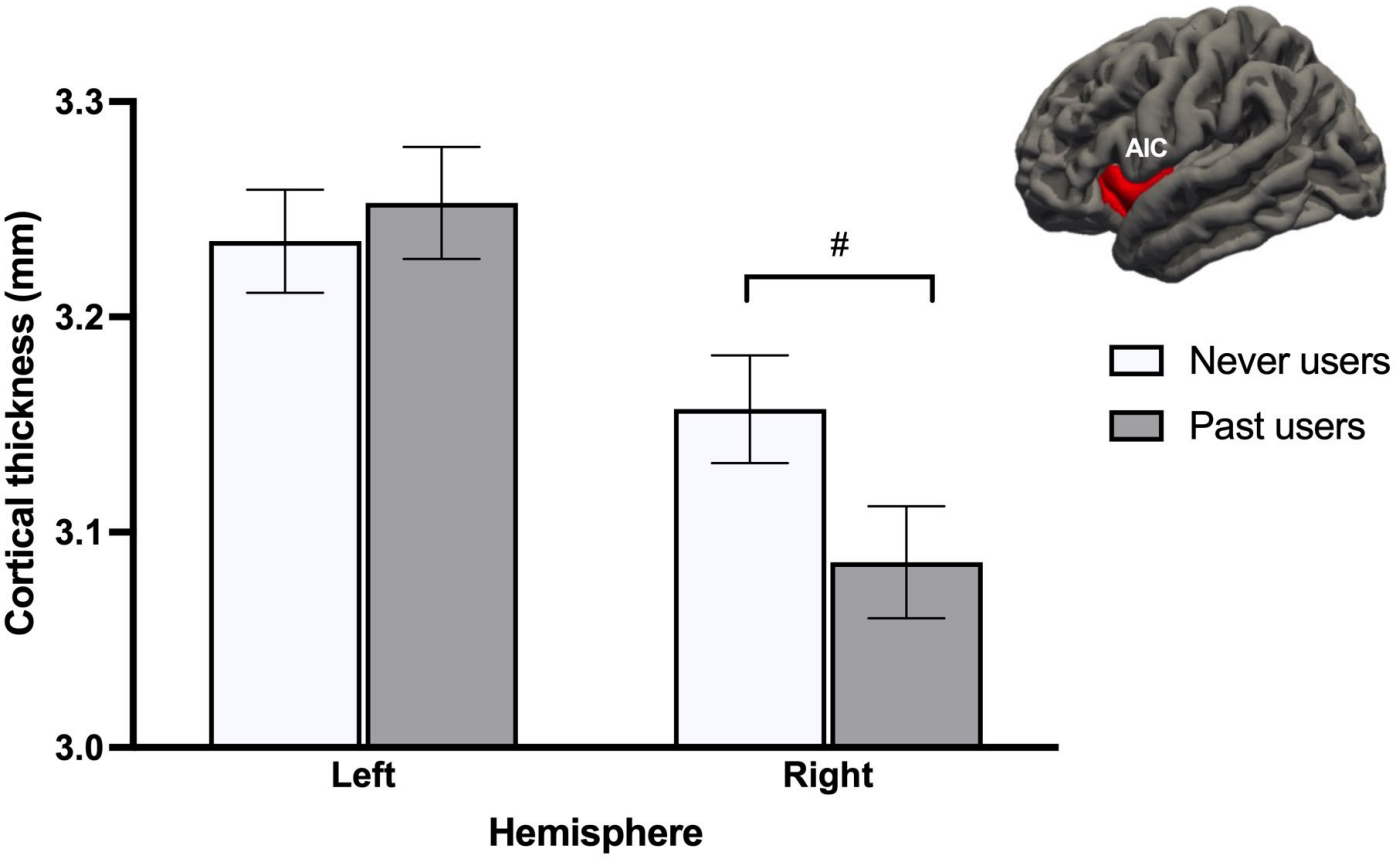
Never users tended to show thicker right anterior insular cortex (AIC) than past users, after adjusting for age and menstrual cycle phase. This difference became significant after adjusting for additional confounds. #*p* <.08.

When looking at both groups of NC women, GMV analyses revealed no effects of Group (past users, never users), Cycle phase (early follicular, pre-ovulatory), or Group x Cycle phase across all ROIs. For CT, a Group x Hemisphere interaction effect was found for the AIC [*F*_(1, 72)_ = 6.40, *p* = .014, *η_p_^2^* = .082, *q*FDR = .054]. Irrespective of cycle phase, never users tended to have a thicker right AIC compared to past users (*p* = .057; **Figure 3**). This difference reached significance (*p* = .039) with the fully adjusted model.

We found no significant clusters in volumetric and thickness WBA. Using VBM, WBA yielded no significant results, though we found a trend for a cluster in the right orbital gyrus for the Group x Cycle phase interaction term [*F*_(1, 72)_ = 11.77, *q*FDR = .072, *k* = 606, MNI_xyz_ = 24, 39, -12]. This cluster fell at *q*FDR = 0.95 with the fully adjusted model.

#### 3.2.3 Are endogenous estradiol and testosterone linked to structural correlates of the fear circuitry? Are those associations specific to men, never users, or past COC users?

**Figure 4:**
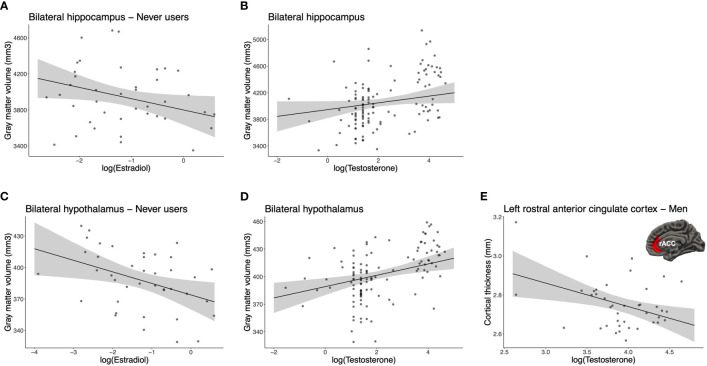
Associations between endogenous sex hormones and brain morphology. For the bilateral hippocampus, **(A)** estradiol was negatively correlated in never users (*p_M_* = .040, fraction missing info = .185) and **(B)** testosterone tended to positively correlate in all participants (*p_M_* = .067, fraction missing info = .078). For the bilateral hypothalamus, **(C)** estradiol was negatively correlated in never users (*p_M_* = .014, fraction missing info = .197) and **(D)** testosterone was positively correlated in all participants (*p_M_* = .002, fraction missing info = .128). **(E)** For the left rostral anterior cingulate cortex (rACC), testosterone was negatively correlated to the cortical thickness in men (*p_M_* = .006, fraction missing info = .021). For illustrative purposes, each scatter plot depicts the imputation dataset being the most similar to the pooled *p*-value of the *post hoc* result. Error bars represent 95% confidence intervals.

To evaluate the role of acute endogenous levels and their potential interaction with our study groups, we conducted Group (3) x Hemisphere (2) x Hormone (E2 or T) GLMs on our imputed and log-transformed dataset. ROI-based GMV analyses revealed a Group x E2 interaction [*F_M_*_(2, 108)_ = 4.22, *p_M_* = .029 (.003 ≤ *p*s ≤.086), *η_p_^2^_M_* = .073, *q*FDR = .203] for the hippocampus. *Post hoc*s showed that bilateral hippocampal GMV correlated negatively with E2 levels (*B_M_* = -124.33, *β_M_* = -.345, *p_M_* = .040, fraction missing info = .185) in never users, specifically. A main effect of T was also trending [*F_M_*_(2, 108)_ = 4.61, *p_M_* = .052 (.007 ≤ *p*s ≤.145), *η_p_^2^_M_* = .041, *q*FDR = .196], where T concentrations tended to positively correlate with the GMV of the bilateral hippocampus in all participants (*B_M_* = 48.58, *β_M_* = .181, *p_M_* = .067, fraction missing info = .078; **Figures 4A, B**).

For the hypothalamus, we found a marginal interaction for Group x E2 [*F_M_*_(2, 108)_ = 2.98, *p_M_* = .079 (.016 ≤ *p*s ≤.233), *η_p_^2^_M_* = .053, *q*FDR = .277]. GMV of the bilateral hypothalamus was inversely correlated to E2 levels in never users (*B_M_* = -13.02, *β_M_* = -.429, *p_M_* = .014, fraction missing info = .197). Also, we found a trend towards a main effect of T [*F*_(1, 108)_ = 4.09, *p_M_* = .056 (.015 ≤ *p*s ≤.151), *η_p_^2^_M_* = .037, *q*FDR = .196], where T concentrations across all groups were positively associated with the bilateral hypothalamus (*B_M_* = 6.15, *β_M_* = .314, *p_M_* = .002, fraction missing info = .128; **Figures 4C, D**).

For CT analyses, we found a marginal Group x Hemisphere x T interaction [*F_M_*_(2, 109)_ = 3.31, *p_M_* = .067 (.007 ≤ *p*s ≤.158), *η_p_^2^_M_* = .058, *q*FDR = .268] for the rACC. When examining *post hoc*s, the only significant result pertained to men, where thickness of their left rACC was inversely associated with T levels (*B_M_* = -.12, *β_M_* = -.421, *p_M_* = .006, fraction missing info = .021; **Figure 4E**). Of note, potential outliers were visually acknowledged. Using a Cook’s distance >.5, we excluded two influential data points and observed that the relationship survived in trend (*B_M_* = -.089, *β_M_* = -.334, *p_M_* = .077, *n* = 38). No other comparison was related to the left or right rACC thickness for T in never and past users.

Sensitivity analyses for all *post hoc*s are further presented in **Supplementary Figures 4**–**8**.

#### 3.2.4 Are exogenous sex hormones linked to structural correlates of the fear circuitry in current COC users? Identifying the most influential factor between salivary EE levels, EE dosage, and progestin androgenicity

**Figure 5:**
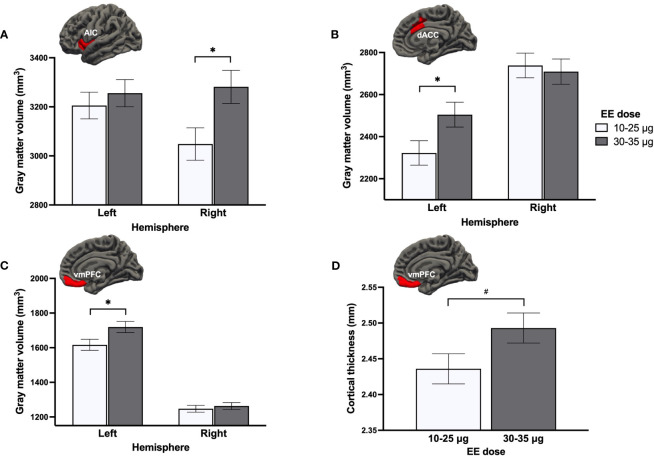
Effect of ethinyl estradiol **(EE)** doses on the gray matter volume of the **(A)** anterior insular cortex (AIC), **(B)** dorsal anterior cingulate cortex (dACC), **(C)** ventromedial prefrontal cortex (vmPFC), and **(D)** the cortical thickness of the vmPFC. *p <.05, #*p* <.08.

In COC users, we performed GLMs for each ROI with Hemisphere (2), EE dose (2), and Salivary EE levels, as well as Hemisphere x EE dose and Hemisphere x EE levels. For GMV, Hemisphere x EE dose interactions were found in the AIC [*F_M_*_(1, 50)_ = 5.08, *p_M_* = .029 (.026 ≤ *p*s ≤.032), *η_p_^2^_M_* = .092, *q*FDR = .098], the dACC [*F_M_*_(1, 50)_ = 4.92, *p_M_* = .031 (.027 ≤ *p*s ≤.035), *η_p_^2^_M_* = .090, *q*FDR = .098], and the vmPFC [*F_M_*_(1, 50)_ = 4.21, *p_M_* = .046 (.042 ≤ *p*s ≤.051), *η_p_^2^_M_* = .078, *q*FDR = .098]. *Post hoc*s showed that women using COCs with a higher EE dose (30-35μg, *n* = 27) exhibited a larger right AIC (*p_M_* = .020), left dACC (*p_M_* = .037) and left vmPFC (*p_M_* = .033) than women using a lower dose of EE (10-25μg, *n* = 28; **Figures 5A–C**). This interaction term was also trending in the rACC [*F_M_*_(1, 50)_ = 3.84, *p_M_* = .056 (.047 ≤ *p*s ≤.066), *η_p_^2^_M_* = .071, *q*FDR = .098], although no *post hoc*s emerged significant (*p_M_* s ≥.101). For CT, a main effect of EE dose was trending in the vmPFC [*F_M_*_(1, 51)_ = 3.46, *p_M_* = .069 (.056 ≤ *p*s ≤.084), *η_p_^2^_M_* = .064, *q*FDR = .276], with 30-35μg doses being marginally associated with thicker bilateral vmPFC compared to 10-25μg doses (**Figure 5D**). Results were similar when adjusting for androgenicity, where higher doses were still associated with greater GMV (though statistical significance was not met for all ROIs).

**Figure 6:**
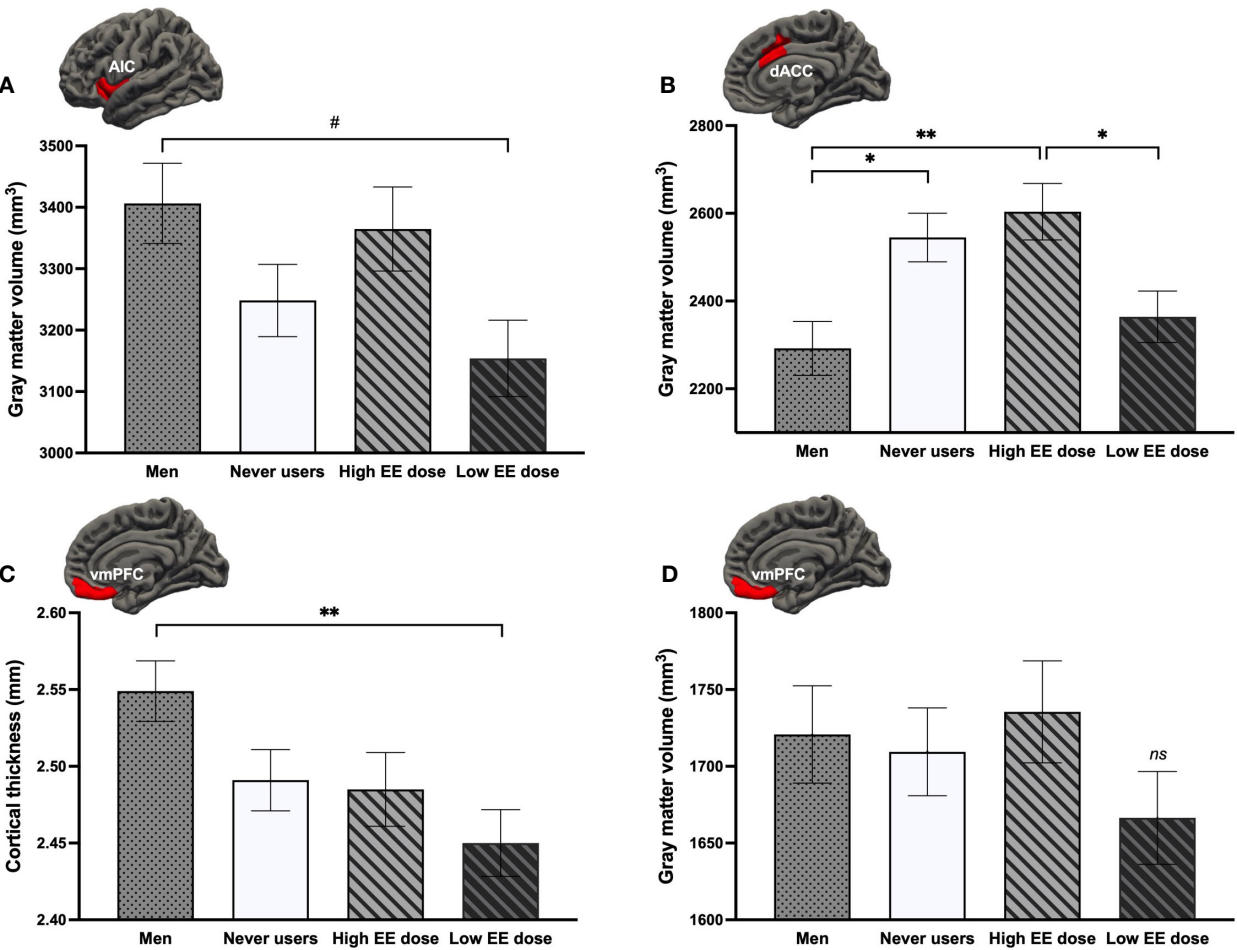
Comparison of current users either taking low or high doses of ethinyl estradiol (EE) with never users and men regarding the **(A)** right anterior insular cortex (AIC) volume, **(B)** left dorsal anterior cingulate cortex (dACC) volume, **(C)** bilateral ventromedial prefrontal cortex (vmPFC) thickness, and **(D)** left vmPFC volume. **p <.01, *p <.05, #*p* <.08. ns, non-significant.

To better understand the directionality of the influence of EE dose, we compared both groups of EE dose users to never users and men on GMV of the right AIC, left dACC, left vmPFC, and CT of the bilateral vmPFC. A main effect of Group was found for the right AIC [*F*_(3, 137)_ = 2.98, *p* = .034, *η_p_^2^* = .061], left dACC [*F*_(3, 137)_ = 5.38, *p* = .002, *η_p_^2^* = .105], and bilateral vmPFC thickness [*F*_(3, 138)_ = 4.03, *p* = .009, *η_p_^2^* = .080] (**Figures 6A–C**). Low EE dose users tended to have a smaller GMV of the right AIC compared to men (*p* = .051). For the left dACC GMV, a region previously shown to be larger in all groups of women (section 3.2.1.), only never users and high EE dose users presented greater GMV than men (*p*s ≤.031), with low EE dose users being most similar to men and having significantly less GMV than high EE dose users (*p* = .037). For the CT of the bilateral vmPFC (where current use of COCs was linked to vmPFC thinning compared to men; section 3.2.1.), this more in-depth investigation revealed that this difference was specific to COC users taking low doses of EE (*p* = .006), with high EE dose users being most similar to never users. Finally, albeit no group effect was found for the left vmPFC (*p* = .434), GMV appeared to be smaller in women using COCs with a lower dose compared to the three other groups (**Figure 6D**).

**Figure 7:**
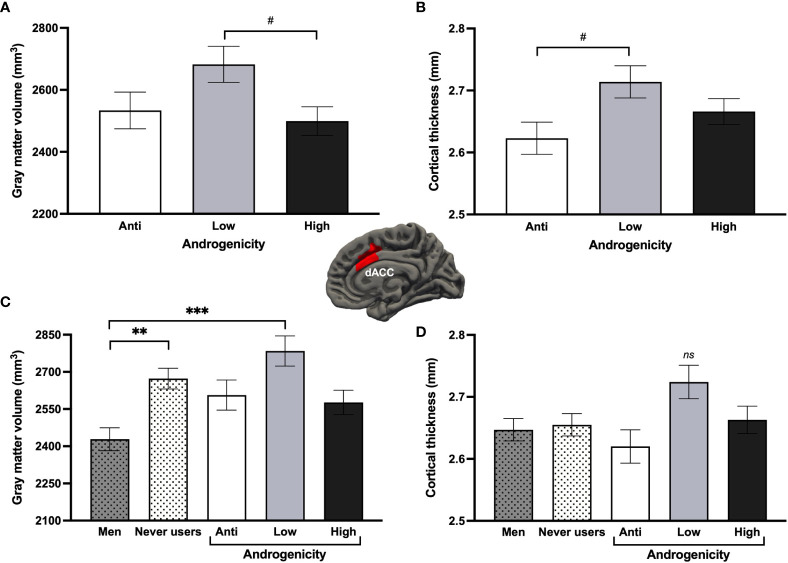
Effects of progestin androgenicity on the dorsal anterior cingulate cortex (dACC). Results are displayed for **(A)** gray matter volume and **(B)** cortex thickness of the three androgenicity groups, as well as additional comparison with never users and past users for **(C)** gray matter volume and **(D)** cortex thickness of this region. ***p <.001, **p <.01, #*p* <.08. ns, non-significant.

Looking at progestin androgenicity, Hemisphere (2) x Androgenicity (3) GLMs revealed no significant effects related to androgenicity. A marginal main effect of androgenicity was found for the dACC GMV [*F*_(2, 57)_ = 3.04, *p* = .056, *η_p_^2^* = .096, *q*FDR = .389] and dACC CT [*F*_(2, 58)_ = 2.95, *p* = .060, *η_p_^2^* = .092, *q*FDR = .241]. Women taking low androgenic COCs (*n* = 18) tended to have greater bilateral GMV than those taking high androgenic COCs (*n* = 27; *p* = .055) and greater bilateral CT than those taking anti-androgenic COCs (*n* = 17; *p* = .055; **Figures 7A, B**). Results remained similar when adjusting for EE dosage. When further comparing androgenicity groups to never users and men for the bilateral dACC, groups significantly differed for the GMV [*F*_(4, 136)_ = 5.73, *p* <.001, *η_p_^2^* = .144], where men exhibited less GMV than never users (*p* = .004; as previously shown above) but also than low androgenic COC users (*p* <.001). Though no significant group effect was found for the dACC CT (*p* = .086), low androgenic COC users appeared to have a thicker bilateral dACC than all groups (*p*s ≥.082; **Figures 7C, D**).

For EE models, no significant clusters emerged for all WBA (volume, thickness, VBM). For androgenicity models, we found significant clusters for CT of the right middle/superior frontal cortex [cluster-wise *p* = .0085, MNI_xyz_ = 21.9, 52.3, 25.6, max = 3.38, size (surface area) = 2976.14mm^2^, *k*_vertices_ = 4928] and GMV of the right caudal middle temporal gyrus [cluster-wise *p* = .023, MNI_xyz_ = 58.9, -54.4, 0.1, max = 3.17, size (surface area) = 946.08mm^2^, *k*_vertices_ = 1875]. T-test *post hoc*s showed that low androgenic COC users had thicker cortex of the right frontal cluster than anti-androgenic and high androgenic COC users, while GMV of the right temporal cluster was larger in anti-androgenic COC users than both low and high subgroups. Both results survived when controlling for EE dose. VBM yielded no significant clusters.

## 4 Discussion

This study examined the modulation of endogenous and exogenous sex hormones on structural properties of key regions implicated in fear expression and regulation. We used rigorous methodology to decipher whether acute or long-term effects of COCs were detectable and if we could document an influence related to menstrual cycle phase, endogenous sex hormone concentrations, and exogenous endocrine factors of COCs. Comparisons of current COC users, past COC users, never users, and men were carried out on surface-based and voxel-based morphometry, as well as with both region-of-interest and whole-brain approaches.

Irrespective of their hormonal contraceptive history, we found that women showed greater GMV than men in the dACC. This ROI-based sex difference aligns with previous reports (146–149) and was further replicated in this study with VBM, among other sex differences found in the right occipital pole, right postcentral gyrus, and left inferior frontal gyrus. These results suggest a predominant role the organizational effects of sex steroids. Indeed, exposure to sex hormones can confer permanent structural modelling of the nervous system when occurring in a specific developmental window (150). Given that COCs are often initiated during adolescence (111, 151–153) and that this developmental stage is considered to be a rich (re-)organizational period (150, 154–156), we can assume that GMV of certain regions like dACC are particularly sensitive to the influence of sex hormones in an earlier developmental stage. This may explain why later endocrine disruption such as via initiation of COCs did not yield anatomical alteration. Naturally, other non-hormonal factors may underlie this finding, such as gender (53). Given that this result survived the adjustment for many socio-cultural factors, we are inclined to lean towards a biological explanation. That said, as we did not measure all relevant gendered confounds, we are unable to support our claim with the current data. Furthermore, we wish to acknowledge the complexity of sex and gender science and the interaction between both dimensions (157). Moreover, it is worth noting that many cluster-corrected findings emerged from SPM’s VBM analysis, though not from FreeSurfer’s surface-based volumetric WBA. Beyond the discrepancy across software, this provides insight into VBM’s sensitivity to sex-sensitive discoveries versus SBM.

The dACC is a region associated with fear expression and appraisal (39, 158). It was found to be involved in the neural signature of trait anxiety, where its volume was reported to be a significant predictor (106). A larger GMV of the dACC was also found to be a discriminant region between drug-naïve anxiety patients compared to healthy controls (159). While the role of the dACC in anxiety appears quite clear, it has also been associated with positive emotion regulation constructs including the use of cognitive reappraisal (160), the ability to identify and describing one’s feelings (161), and effortful control (162, 163). These conflicting results may stem from the broad role of dACC in emotion processing and the fact that these complex mental states are sustained by large networks rather than a single brain region. They can also be reconciled with regard to paradoxical traditional women-oriented stereotypes, where women are expected to carry heavier household responsibilities, plan ahead, have emotional intelligence, and care and worry about others (164–166).

Our results also showed a reduced CT of the vmPFC in current COC users compared to men. This aligns with Petersen et al. (57, 58) who also showed a reduction of CT in prefrontal areas, although they found significant disparities between NC women and COC users and did not have a group of men for comparison (57). Previous studies have reported thicker vmPFC in men compared to women (167, 168), however, this difference has not been replicated enough to support a reliable sex difference (169–171). Thus, a specific comparison of COC users and men may elucidate the mixed results regarding a potential sex difference of the vmPFC thickness. Moreover, the vmPFC is consensually viewed as a critical node for fear regulation (13, 38, 39, 158). Thicker vmPFC has been associated with greater fear extinction learning and recall (172, 173) and lesser fear generalization (174), as well as resilience following trauma exposure (175) and remission in treatment-naïve patients with obsessive-compulsive disorder (176). Reduced vmPFC thickness is thought to reflect top-down inhibition deficits, particularly of amygdala reactivity (177, 178). Interestingly, poorer extinction recall has been reported in COC users compared to NC women in a high E2 state (30). COC users also showed reduced responsiveness to exposure therapy compared to NC women irrespective of their E2 levels (8, 179). In light of these findings, our vmPFC result suggests that COCs may confer a risk factor for extinction deficits during their current use but not after their discontinuation. Indeed, it is well known that women are more susceptible to suffering from fear-related psychopathologies than men, including anxiety and stress-related disorders (3, 16). Given our results that men have smaller dACC volume than women and thicker vmPFC than COC users, these findings may represent structural vulnerabilities to psychopathologies that predominantly affect women. Specifically, a larger dACC could represent a female predisposition to fear promotion, whereas COC use could exacerbate this vulnerability by potentially inducing a thinning of a fear-inhibiting region such as the vmPFC. However, as tempting as it may be to settle on this conclusion, caution must be exercised when interpreting interrelations between brain morphology and behavioral or psychological data. Despite the high reliability of structural imaging (180), the validity of brain-behavior associations has been criticized (181, 182).

Interestingly, no lasting effects of COC use were detected when comparing the four groups. The sole difference pointing towards such effects was in the right AIC, where never users had a greater CT than past users when controlling for the ‘here and now’ influence of the menstrual cycle. The right AIC has been found to be engaged in bodily-arousing anxiety-related processing such as heartbeat awareness, sympathetic nervous system activities, and unpredictable aversiveness (183–186). Knowing that its volume has been negatively associated with reactivity to uncertain threat (187), thinner CT in past users could possibly be interpreted as deleterious. Most importantly, our overall findings support the reversibility of the impact of COCs on brain morphology, especially on vmPFC thickness. Yet, our conceptualization of the past user group may not have been optimal for detecting long-term effects. Indeed, as the literature provided little guidance, we used an arbitrary (yet stringent) criterion of 12 months for a minimal duration of use and time since discontinuation. Greater variability for cessation duration (i.e., setting a criterion to a shorter elapsed time) would have allowed a finer investigation of lasting effects. Therefore, our results prompt future research to explore the reversibility of COC use within the first year of discontinuation. In addition, pooling all past users together may have camouflaged potential long-term effects. Given that adolescence is a sensitive period for brain development and that OC use at this age has been linked to a higher vulnerability to depression (111, 188–191), OC parameters (e.g., duration of use and age of onset) may be other relevant and possibly more sensitive factors for studying durable effects (192). Of note, our team is currently investigating the effects of these parameters.

For endogenous sex hormone concentrations, we observed similar patterns for the hippocampus and hypothalamus, where these two structures are highly susceptible to the influence of sex hormones (193–195). However, the inverse relationships found for E2 in never users were quite surprising and opposed the scientific consensus on the trophic effects of E2 on the hippocampus (45–48). Considering our unique methodological approach, our hormone-brain associations should be interpreted cautiously and require replication. Statistical significance varied considerably across imputations, as observed by wide *p*-value ranges and the ‘fraction missing information’ metric. The latter indicates the variance proportion pertaining to the multiple imputation procedure. Specifically, in our study, the variation of one dataset to another amounted to nearly 20% and was the result of a high non-detection rate using LC-MS/MS. Importantly, LC-MS/MS provides better assay validity than immunoassay methods for salivary E2 and P (especially for low ranges) and is on its way to becoming the gold standard in endocrine measurement (196–199). Our study shows that for a normative adult population (i.e., without pathology, naturally-circulating and low sex hormone levels), data from LC-MS/MS are relevant and valid for categorization purposes (mean comparisons). However, according to our study, the main drawback of this method relates to statistics based on the continuous nature of sex steroid levels. Knowing that saliva accounts for free (unbound) steroids and represents less than 5% of total serum concentrations (200, 201), we recommend the use of serum samples for future studies conducting correlational analyses on steroid data obtained via LC-MS/MS. Visibly, the future of LC-MS/MS is promising and efforts in refining actual methods for studying sex steroids are within reach. Improvement in detection rates would certainly allow the scientific community to better grasp the influence of endogenous sex hormones on the brain.

For exogenous steroids, we first determined that EE dose (rather than circulating EE levels) modulated cortical fear-related ROIs in COC users. This observation is fairly unsurprising, as we expect brain tissues to adapt over a certain period of time (202). For instance, we expect this adaptation with constant and chronic exposure to EE dose, rather than with the rapid fluctuations inherent to pharmacokinetics. Indeed, GMV of the right AIC, left dACC, and left vmPFC were significantly larger in COC users using higher doses of EE compared to lower doses. We were able to contextualize the direction of this effect by comparing these groups to men and never users, revealing the atrophic effects of a lower EE dose rather than the trophic effect of a higher EE dose. This also allowed us to refine the result regarding CT of the vmPFC in current COC users, where thinning was driven by lower doses of EE. Interestingly, the potency of EE is approximately two times greater than that of E2 on the estrogen receptor alpha (ERα), while mixed findings have been reported for the estrogen receptor beta (ERβ) (85, 203, 204). Both ERs have been detected in the cortex (205, 206), with a larger proportion of ERα reported in the vmPFC (28). Given that endogenous E2 is suppressed by any kind of COCs, one could hypothesize that only low EE intake (i.e., 10-25μg) may lead to underactivation of ERα (and potentially ERβ to a lesser degree) and therefore, prevent cortical trophic effects of estrogenic activity (207, 208). Though we were unable to empirically explore this option, the impact of EE dosage on brain volumes may follow a curvilinear trajectory where higher EE doses (e.g., 50 μg) may induce atrophic effects. Inverted-U relationships of E2 have been reported on fear extinction (209) and hippocampal activity (210). Despite its speculative nature, this hypothesis could be further tested in animal models to deepen our understanding of basic endocrinology knowledge. We however strongly advise against testing this hypothesis in women considering the known risk of much higher EE doses on safety and tolerability of COCs (26, 211). Yet, based on our results, we propose that a hypoestrogenic state may only occur when low endogenous E2 is combined with low EE intake. Conversely, higher EE doses may provide adequate ERα binding to simulate moderate-to-high estrogenic activity, similar to NC women. Recent reviews in the field have highlighted the importance of disentangling OC mechanisms with regard to their endogenous (i.e., inhibition of the HPG axis) versus exogenous actions (i.e., intake of synthetic molecules) (84, 85, 212, 213). While this line of interpretation remains novel, our results provide insight into how COC users may be under a hypoestrogenic state when using regimens containing lower EE doses.

In contrast, our investigation on progestin androgenicity did not yield convincing effects on the fear circuitry. We reported a possible inverted-U relationship in the dACC, where low androgenic activity tended to show trophic influence over this cortical region. This curvilinear relationship appears to be more largely spread and stronger in the upper part of the frontal cortex, as observed by the means of an exploratory WBA. However, even after statistically controlling for EE dosage, these findings could still potentially be confounded by higher EE doses in low androgenic COC users. Due to the small sample size for these models, it is difficult to clearly depict the impact of androgenicity on the fear circuitry. To do so, it would be essential to conduct analyses on a larger sample and to focus on the interaction between EE dose and androgenicity.

We measured two phenotypes of the GM that have been previously linked to OC use, namely GMV and CT (59–61). In our study, some results pertaining to cortical ROIs were found for GMV, CT, or both. On the one hand, effects specific to CT (e.g., thinner vmPFC in current users) or found for both phenotypes (e.g., marginal influence of androgenicity on the dACC) suggest changes in cell restructuring among cortical layers. On the other hand, effects in GMV but not in CT (e.g., sex difference in the dACC) suggest that volumetric alterations are consequent to other properties of the GM such as folding, surface area, or an interplay between CT, folding and/or surface area. Further investigation of various GM phenotypes could help singularize which aspect of GM is particularly influenced by volumetric findings.

For the ROI approach, we opted for an uncorrected significance threshold for the multiple ROIs, even though *q*FDRs were presented for completeness. As multiple testing is an important issue in MRI studies, it is typically handled using FDR or Bonferroni corrections. Yet, when applied to ROIs as opposed to WBA, this procedure is highly dependent on the number of ROIs and may give the false impression of non-significant results (93). Given the strong *a priori* rationale for ROIs defined in our study, we deemed that all (uncorrected) results were relevant. By looking at effect sizes and *q*FDRs, our findings clearly show that the results of medium-to-large effect size did not survive the FDR adjustment of *q* <.05 for ROIs of *k_GMV_* = 7 and *k_CT_* = 4. Despite filtering out results with the smallest *p*-values, this highlights the weakness of *a posteriori* corrections (i.e., inflation of false negative rate) for ROI-based analyses.

Throughout the discussion, few limitations of the present study were identified. Although the non-randomized cross-sectional design was informative considering the novelty of this research, it also warrants caution when making causal inferences. Moreover, the inclusion of other menstrual cycle phases (e.g., luteal phase) would have allowed for a more holistic portrait of NC women. Relatedly, our study is characterized by low external validity due to stringent sample criteria (e.g., no current or previous psychiatric/physical diagnosis). Thus, even if we were able to rule out any ‘survivor effect’ among current users (by having a group of past users), we had a very pure sample of individuals that may be distinct from other subgroups. Therefore, generalization of the results is limited and could explain discrepancies with the literature.

In addition, we identified several future directions and recommendations for the field. Above, we highlighted the relevance of exploring the following avenues: the relative role of sex and gender in terms of OC use and brain correlates, the lasting-but-reversible OC effects in women having recently stopped using OCs, and other OC parameters relevant to the discovery of long-term effects including duration of use and age of onset. Exploration of other structural properties and replication would also be of great interest. Moreover, replicating this study with other imaging methods (e.g., functional MRI) during a fear conditioning and extinction task would certainly allow for a better understanding of the current anatomical findings.

While our research does not have a direct clinical focus, it nonetheless contributes to advancing our fundamental understanding of the structural brain correlates of COC use. Our aim is to stimulate applied research by providing valuable insights and knowledge in the field of psychoneuroendocrinology. We first recommend evaluating the dose-dependent EE effects on cortical GM, then the clinical significance of our results, especially the atrophic effect of low EE doses. While our data may encourage prescribing COCs containing 30-35μg of EE for women presenting emotion-dysregulation psychopathologies, future research should prospectively investigate (in first-time COC users) the clinical effect size of such atrophy on fear-related symptomatology.

## 5 Conclusion

As OC use is so widespread, it is important to better understand its current and long-term effects on brain anatomy and emotional regulation. Our study demonstrated that COC intake can affect fear-related brain morphology, but that these effects can be reversible over time. Studying the brain circuitry underlying fear regulation and its modulation by endogenous and exogenous sex hormones could deepen our understanding of the etiology and maintenance of fear-related psychopathologies predominantly affecting women such as anxiety disorders and post-traumatic stress disorder.

## Data availability statement

The raw data supporting the conclusions of this article will be made available by the authors, without undue reservation.

## Ethics statement

The studies involving human participants were reviewed and approved by the research ethics board of the Centre intégré universitaire de santé et de services sociaux de l’Est-de-l’Île-de-Montréal. The patients/participants provided their written informed consent to participate in this study.

## Author contributions

AB and MFM contributed to the conception and design of the study. AB, LMD, and AMT managed data collection. AB organized the database, performed the statistical analyses, and wrote the first draft of the manuscript. AMT wrote a section of the manuscript. All authors contributed to manuscript revision, read, and approved the submitted version.
