# Supplementary material for: Morphologic alterations of the fear circuitry: the role of sex hormones and oral contraceptives
Source: Front Endocrinol (Lausanne). 2023 Nov 7;14:1228504. doi: 10.3389/fendo.2023.1228504 (PMC10661904; doi:10.3389/fendo.2023.1228504)
Supplement: Supplementary file 1 [file Image_1.pdf]

Supplementary Material

Morphologic Alterations of the Fear Circuitry: The Role of Sex Hormones and Oral Contraceptives

Alexandra Brouillard[1,2*], Lisa-Marie Davignon[1,2], Anne-Marie Turcotte[3], Marie-France Marin[1,2]

[1]Research Center of the Institut universitaire en santé mentale de Montréal, Montreal, QC, Canada
[2]Department of Psychology, University of Quebec in Montreal, Montreal, QC, Canada
[3]Department of Medicine, University of Montreal, Montreal, QC, Canada

*** Correspondance:**
Alexandra Brouillard
brouillard.alexandra@courrier.uqam.ca

1 Supplementary Figures

1. Visualization of voxel-based morphometry clusters emerging between the four groups using a whole-brain approach, adjusted for age and scaled for total intracranial volume.

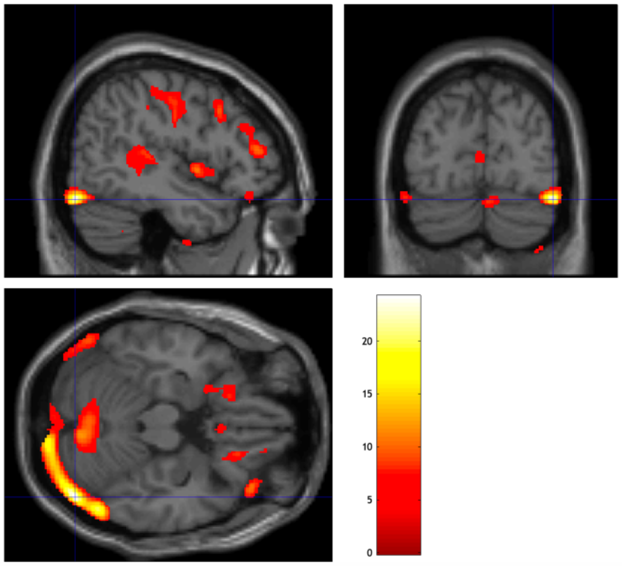

Supplementary Figure 1. Crosshairs are positioned at the peak voxel of the right occipital cluster. Parts of the right dlPFC, middle frontal, and superior temporal clusters are displayed on the sagittal plane. The transverse plane also displays a part of the right orbital cluster.

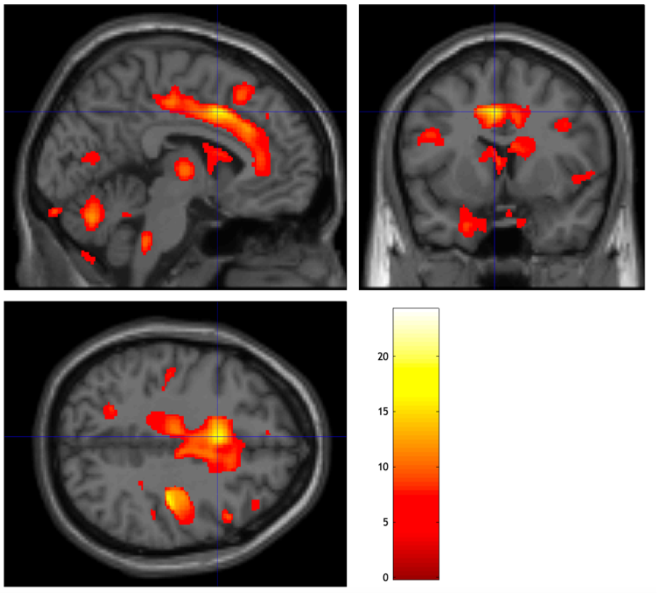

Supplementary Figure 2. Crosshairs are positioned at the peak voxel of the dACC cluster. Parts of the right thalamus, right cerebellum, and left cuneus clusters are displayed on the sagittal plane. The postcentral cluster is partly depicted on the transverse plane, while the coronal plane partly shows the left temporal pole/parahippocampal cluster. The right middle frontal cluster is visible on the coronal and transverse planes.

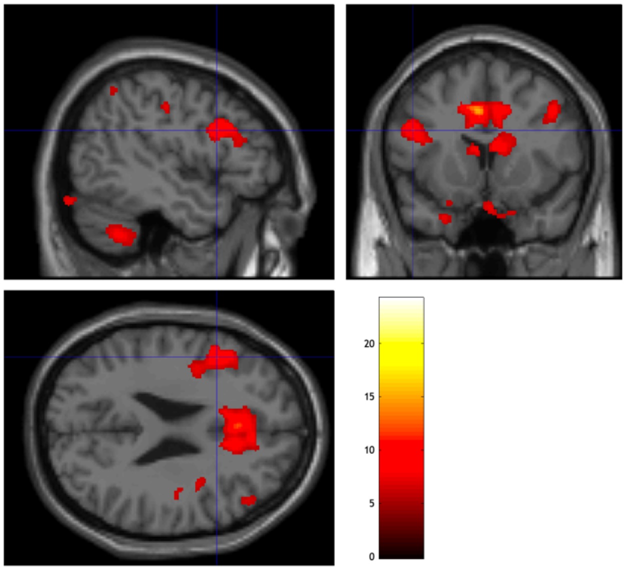

Supplementary Figure 3. Crosshairs are positioned at the peak voxel of the left Opercular inferior frontal cluster. Parts of the right middle frontal and dACC clusters are displayed on the coronal and transverse planes. The sagittal plane also shows a part of the left cerebellum cluster.

2. Sensitivity analyses were conducted on post hocs reported in section 3.2.3. of the manuscript. Raw hormone levels (pg/mL) were categorized to further examine the relationships obtained in the main analyses.

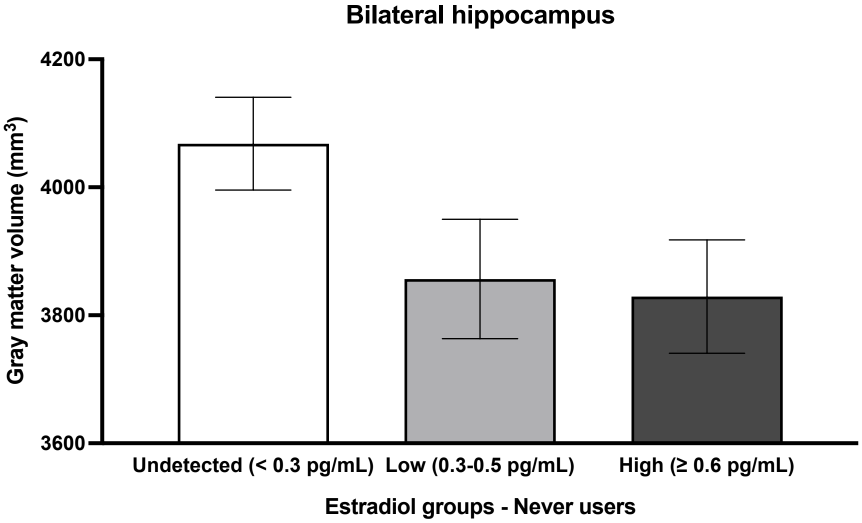

Supplementary Figure 4. Main effect of estradiol concentrations in never users on gray matter volume of the bilateral hippocampus. The figure was adjusted for age, total intracranial volume, and log(T) levels. Error bars are SEM. In a univariate ANCOVA, the group effect was at $p_M = .106$ ($.015 \leq p$s $\leq .188$). Group sizes were as follows: Undetected $n = 18$, Low $n = 10$, High $n = 12$.

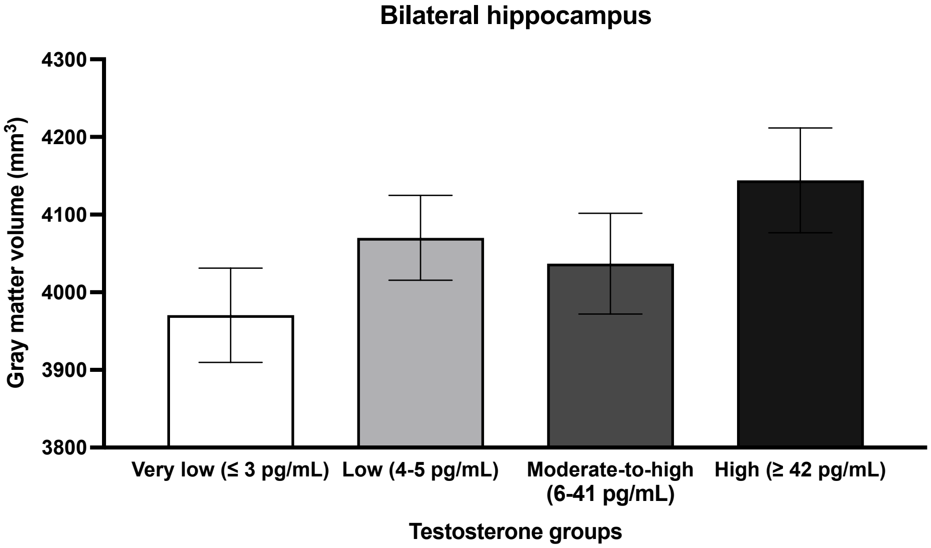

Supplementary Figure 5. Main effect of testosterone concentrations on gray matter volume of the bilateral hippocampus. As no interactions with groups were found, never users, past users, and men were combined here. The figure was adjusted for age, total intracranial volume, and log(E2) levels.

Error bars are SEM. In a univariate ANCOVA, the group effect was at $p_M = .323$ ($.280 \leq ps \leq .351$). Group sizes were as follows: Very low $n = 30$, Low $n = 34$, Moderate-to-high $n = 23$, High $n = 30$.

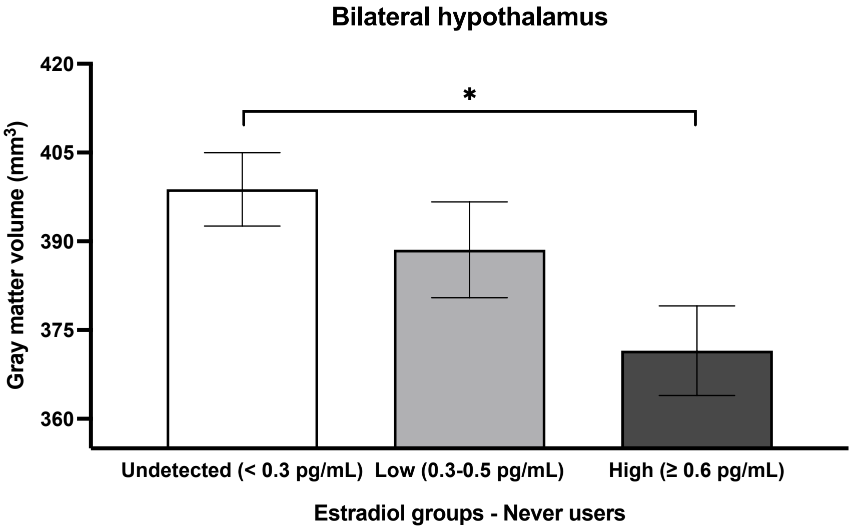

Supplementary Figure 6. Main effect of estradiol concentrations in never users on gray matter volume of the bilateral hypothalamus. The figure was adjusted for age, total intracranial volume, and log(T) levels. Error bars are SEM. In a univariate ANCOVA, the group effect was at $p_M = .041$ ($.019 \leq ps \leq .066$). Group sizes were as follows: Undetected $n = 18$, Low $n = 10$, High $n = 12$.

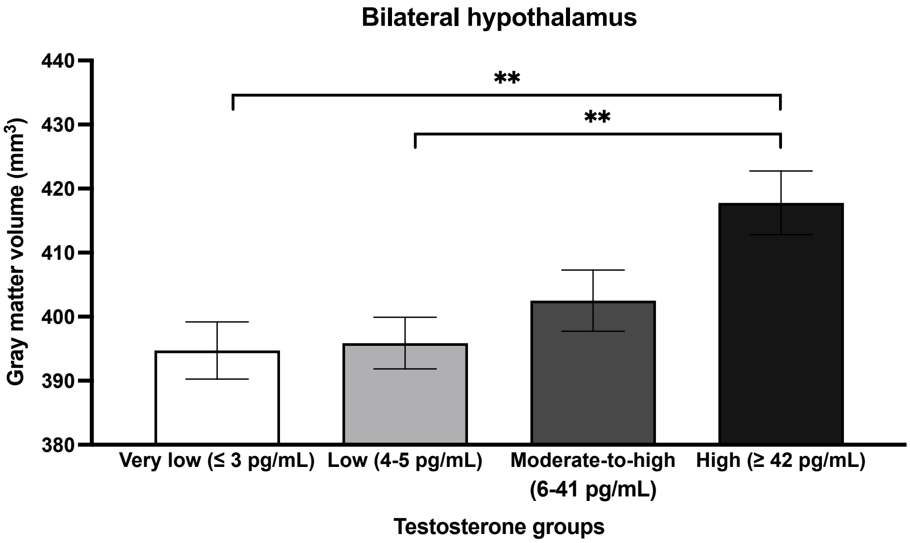

Supplementary Figure 7. Main effect of testosterone concentrations on gray matter volume of the bilateral hypothalamus. As no interactions with groups were found, never users, past users, and men are combined here. The figure was adjusted for age, total intracranial volume, and log(E2) levels. Error

bars are SEM. In a univariate ANCOVA, the group effect was at $p_M = .008$ ($.006 \le p\text{s} \le .013$). Group sizes were as follows: Very low $n = 30$, Low $n = 34$, Moderate-to-high $n = 23$, High $n = 30$.

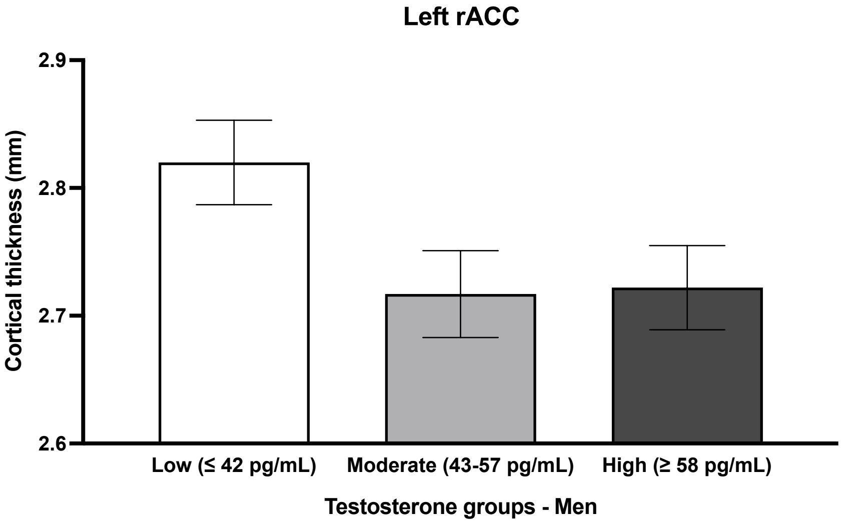

Supplementary Figure 8. Main effect of testosterone concentrations in men on cortical thickness of the left rostral anterior cingulate cortex (rACC). The figure was adjusted for age, and log(E2) levels. Error bars are SEM. In a univariate ANCOVA, the group effect was at $p_M = .054$ ($.042 \le p\text{s} \le .062$). Group sizes were as follows: Low $n = 13$, Moderate $n = 13$, High $n = 14$.
